# The danger theory: 20 years later

**DOI:** 10.3389/fimmu.2012.00287

**Published:** 2012-09-17

**Authors:** Thomas Pradeu, Edwin L. Cooper

**Affiliations:** ^1^Department of Philosophy, Paris-Sorbonne University and Institut Universitaire de FranceParis, France; ^2^Laboratory of Comparative Neuroimmunology, Department of Neurobiology, David Geffen School of Medicine, University of California, Los AngelesLos Angeles, CA, USA

**Keywords:** danger, danger signals, immunity, inflammation, innate immunity, cancer, tolerance, transplantation

## Abstract

The self–non-self theory has dominated immunology since the 1950s. In the 1990s, Matzinger and her colleagues suggested a new, competing theory, called the “danger theory.” This theory has provoked mixed acclaim: enthusiasm and criticism. Here we assess the danger theory *vis-à-vis* recent experimental data on innate immunity, transplantation, cancers and tolerance to foreign entities, and try to elucidate more clearly whether danger is well defined.

## Introduction

The self–non-self theory has dominated immunology for 60 years (Burnet and Fenner, 1949; Burnet, 1959, 1969; Tauber, 1994). More recently, Polly Matzinger suggested a rival theory, called the “danger theory” (Matzinger, 1994, 2001, 2002, 2007, 2012; Gallucci et al., 1999; Anderson and Matzinger, 2000a; Gallucci and Matzinger, 2001; Seong and Matzinger, 2004; Matzinger and Kamala, 2011). This theory claims that immune responses are triggered by “danger signals,” or “alarm signals,” released by the body's own cells (Matzinger, 1994, 2001). According to the danger theory every immune response is not due to the presence of “nonself” (i.e., genetically foreign entities), but to the emission, within the organism, of “danger signals.”

The danger theory aroused much enthusiasm both in scientific journals and in the lay press, but it also provoked reservations (Janeway et al., 1996; Silverstein and Rose, 1997; Vance, 2000; Greenspan, 2007). Because of its pervasiveness some danger theorists have compared the danger theory to the 16th century “Copernican Revolution,” when a heliocentric view of the solar system replaced the geocentric view (Fuchs et al., 1996), provoking irony by some immunologists (Vance, 2000). Because the debate can be more rational, less passionate, today, we will assess the danger theory *vis-à-vis* experimental data, almost 20 years after its first formulation. We will focus on analyzing specific problems, especially the definition of “danger,” the molecular characterization of “danger signals” or “damage signals,” and how the danger theory may help clarify innate immune responses, cancers, transplantation and immune tolerance to exogenous entities (see Box 1).

Box 1 **The main challenges for the danger theory.**

- How should “danger” be defined?
- How should “danger” be compared with “alarm” and “damage”?
- What are molecular traits of “danger signals” or “damage signals”?
- Will the danger theory explain key phenomena such as innate immunity, cancers, transplantation, and tolerance to exogenous entities?
- Does the danger theory offer an analytical *explanation* for immune responses?

## The danger theory: roots and principles

The danger theory was explicitly a critique of how immunologists have been trained, namely, within the self–non-self theory (Matzinger, 1994, 2002). According to the self–non-self theory, an immune response is triggered against all foreign (“nonself”) entities, whereas no immune response is triggered against the organism's own constituents (“self”) (Burnet, 1962, 1969). For Matzinger, despite the evolution of the self–non-self theory between the 1960s and the 1990s, today's immunologists still think of the immune system within this framework, even though this theory may be interpreted as fundamentally flawed.

Against the self–non-self theory, the danger theory claims that self constituents can trigger an immune response, if they are dangerous (e.g., cellular stress, some autografts, etc.); and non-self constituents can be tolerated, if they are not dangerous (e.g., the fetus or commensal bacteria) (Matzinger, 1994, 2002). According to Matzinger and colleagues, the proper opposition to determine why an immune response is triggered is the presence or absence of danger, not exogenous vs. endogenous characters of any entity under consideration.

Doubts can be raised about the novelty of this conception. First, clearly Matzinger elaborated on Janeway's view, based on distinction between “infectious nonself” and “noninfectious self” (Janeway, 1989, 1992). According to Janeway, effector innate immune responses are due to pathological foreign entities (“infectious nonself”) in the host. Janeway proposed that antigen-presenting cells (APCs) evolved to interact with widespread natural microbial patterns or “pathogen-associated molecular patterns” (PAMPs), e.g., lipopolysaccharide (LPS). APCs do not recognize “nonself”; instead, they recognize foreign patterns that are highly conserved throughout evolution. This legacy from Janeway to Matzinger is plausible, but Matzinger emphasized differences between Janeway's view and her's (Matzinger, 2001, 2002). Janeway crucially references to the exogenous nature of rejected entities, whereas Matzinger claims the necessity to fully abandon this perspective. Matzinger asserts that immune responses are not triggered by non-self, but by “endogenous cellular alarm signals from distressed or injured cells.” (Matzinger, 2002: 302; see also Matzinger, 2001: 7). Even more explicitly, Matzinger writes: “the ‘foreignness’ of a pathogen is not the important feature that triggers a response, and ‘self-ness’ is no guarantee of tolerance (Matzinger, 2002: 302).” Table 1 illustrates the differences between theories of self–non-self, infectious non-self, and danger.

**Table 1:** **Predictions made by theories of self–non-self, infectious non-self, and danger (after Matzinger, 2002)**.

**Characterization of the entity**			**Theories**		
**Self/Non-self**	**Danger**	**Pathogen associated molecular patterns (PAMPs)**	**Self–non-self theory**	**Infectious non-self theory**	**Danger theory**
Self	Not dangerous		No response	No response	No response
	Dangerous		No response	No response	Response
Non-self	Not dangerous	No PAMPs	Response	No response	No response
		With PAMPS	Response	Response	No response
	Dangerous	No PAMPs	Response	No response	Response
		With PAMPS	Response	Response	Response

According to the classic self–non-self theory (e.g., Burnet, 1969), only non-self entities trigger an immune response. The infectious non-self theory (Janeway, 1989) states that only infectious non-self, i.e., entities that express pathogen-associated molecular patterns (PAMPs) trigger an immune response, through the activation of antigen-presenting cells (APCs). The danger theory offers different predictions by saying that what triggers an immune response is not “foreigness,” but the release of “alarm signals” by damaged tissues (Matzinger, 2002).

Another critique, coming from historians of immunology (Silverstein, 1996), proposes that Matzinger's view is close to that of Metchnikoff and Ehrlich (Metchnikoff et al., 1893; Ehrlich, 1957). Indeed, both had insisted on the importance of inflammation and damages in immunity (Tauber and Chernyak, 1991). Matzinger and colleagues acknowledge the influence of Ehrlich's thought (Fuchs et al., 1996). We can conclude from these critiques that the danger theory is not utterly new, and therefore that it is probably exaggerated to present it as a “Copernician revolution” (Fuchs et al., 1996). We, however, are more interested in the conceptual and experimental adequacy of the danger theory than in its originality. After all, if Metchnikoff and Ehrlich were right and Burnet was wrong, it is important to demonstrate this claim on the basis of recent experimental data.

## Is the concept of “danger” well defined?

An outstanding feature of the danger theory concerns its proponents' aim to determine an adequate *criterion of immunogenicity*, that is, a clear and testable answer to what triggers an immune response. Danger theorists avoid the all too easy “solution” adopted by many: advocating that immune responses are multifactorial, complex, and contextual. Immune responses undoubtedly are just so, but what immunologists demand is a suitable explanatory and predictive framework to design and conduct research (Pradeu and Carosella, 2006; Pradeu, 2012). The critique of Anderson and Matzinger (2000b,c) at other conceptions clearly illustrates this idea. Moreover, Matzinger and colleagues seek a well-expressed and testable explanation of how an immune response is triggered. We believe that here lies one of the crucial scientific qualities of the danger theory.

To assess the validity of the danger theory, it seems essential to define precisely what is a “danger,” which seems difficult. Matzinger and her colleagues use several terms, that they apparently interpret as equivalent, or necessarily correlated: “danger,” “damage,” “stress,” “injury,” “necrosis,” “inappropriate (/nonphysiological/bad) cell death,” etc. (e.g., Matzinger, 2002, 2007). Yet one can doubt that these terms are synonymous since, for instance, a cell can die from necrosis without causing damage to the organism's tissues. Moreover, “danger” is often excessively anthropomorphic (see, for example, Matzinger, 2007) or teleological (Silverstein and Rose, 1997, 2000). For example, an organ transplant is not “dangerous” but rather useful for the receiver, yet Matzinger proposes that it is dangerous because the surgeon's gesture damages the patient (Matzinger, 2002); in this case, it is not clear what counts as a “danger” for the organism. Furthermore, how does the notion of “danger” explain the immune responses to innocuous antigens such as allergens or food antigens? According to one answer, the immune system inappropriately “sees” these as dangerous, even though they are not “really” dangerous; this exposes the circular logic of the thesis. Thus, it is often unclear what a “danger” is for a cell or a tissue, and how cells and tissues can “perceive” that something is dangerous, supporting this conundrum.

By contrast, Matzinger and colleagues' theory is much more precise if its central claim is that every immune response is due to *damages* to the organism's cells or tissues. Indeed, it is easier to define what a “damage” is (for an organism, a tissue or a cell) than what a “danger” is. In fact, this is the interpretation that Matzinger proposes when she describes the molecular details of her theory (e.g., Matzinger, 1994, 2002; Anderson and Matzinger, 2000a). As Matzinger suggests (Matzinger, 2002), the claim that immune responses were due to “danger” was merely a theoretical suggestion, while the idea that they are due to “damages” has led to several experimental investigations. Therefore, in order to assess the “danger theory,” the main concern is to define “damage” signals.

## From “danger” to “damages”: the molecular identification of “damage signals”

**Figure 1:**
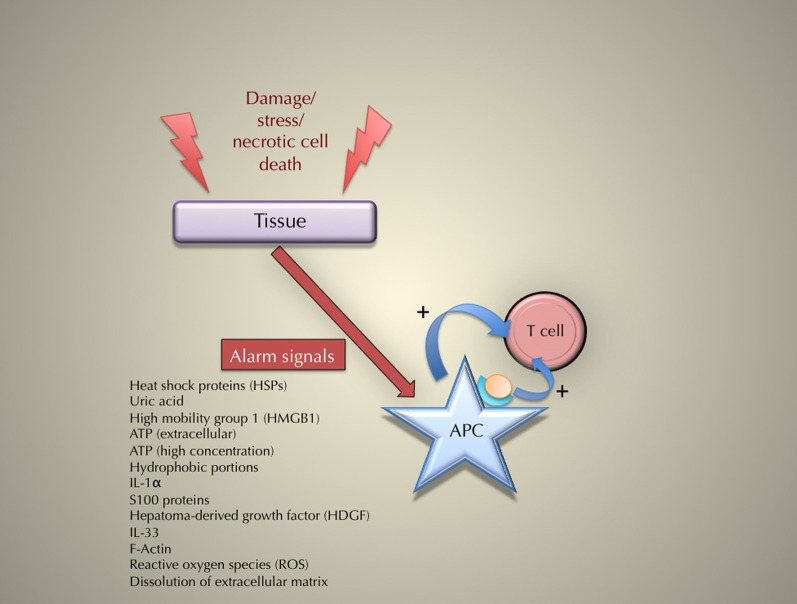
**The principle of the triggering of an immune response according to the danger (or “damage”) theory**.

Matzinger's theory is both clearer and more testable if its main statement proposes that immune responses are due to tissue damages, rather than “danger.” Thus, we submit that the name “damage theory” (rather than “danger theory”) may be more appropriate. Figure 1 sums up the general principle for the triggering of an immune response according to the “damage” theory.

The identification of “damage” signals or molecules is difficult. Here again, Matzinger and colleagues offer distinct suggestions. Thus, cellular stress, heat shock proteins (HSP), interferon-α, interleukin-1 β, uric acid, etc., have been suggested as “damage” signals. Alarm signals may be due to an endogenous or an exogenous damage to tissues (Brown and Lillicrap, 2002), but it is about the former that the danger theory really innovates, and therefore we will focus here on these endogenous signals. What is needed, we believe, is a list of these “damage” signals [see Table 2 for a possible list, and below section “Recently described damage signals” for an up-to-date list], and, even more importantly, criteria to determine whether or not to include a molecule in this list (more on this in sub-section “Recently described damage signals” as well). Many data suggest a close association between an immune response and cellular or tissue damage. Here we examine in detail several cases that illustrate this association. After examining damage signals described by Matzinger and colleagues [cellular stress, heat-shock proteins (HSPs), and necrotic cell death], we turn to signals recently identified by other investigators.

**Table 2:** **Endogenous danger signals according to Matzinger and colleagues (after Gallucci and Matzinger, 2001)**.

**Signals**
CD40-L
TNF-α, IL-1β, IFNα
Intracellular nucleotides: ATP, UTP
Long unmethylated CpG sequences
Heat shock proteins (HSP)
Reactive oxygen intermediates
Vasoactive intestinal peptide (VIP)
Metalloproteinase-9
Degradation products of heparan sulfate
Small breakdown products of hyaluronan

### Cellular stress and immune responses

A first suggestion made by Matzinger and colleagues is that danger signals are in fact signals of “stress” (e.g., Gallucci et al., 1999). According to this proposal, when a cell is stressed, even in the absence of any foreign substance, it emits molecules that activate APCs. Dendritic cells are powerful APCs that must be activated to initiate an immune response. Without any foreign substances, dendritic cells may be activated by endogenous signals. These may be received from cells that are stressed, either by viral infection or cell death by necrosis, whereas these signals are not emitted by healthy or apoptotic cells. In experimental situations if injected *in vivo* with an antigen, any endogenous activating substances can function as natural adjuvants. These may stimulate a primary immune response, which may include natural initiators of transplant rejection, spontaneous tumor rejection, and classes of autoimmunity (Gallucci et al., 1999).

### Heat-shock proteins and immune responses

A second, related, possibility suggests that damage signals consist of HSPs (e.g., Matzinger, 1998; Asea et al., 2000; Gallucci and Matzinger, 2001; see also Wallin et al., 2002). HSPs, described starting in the 1970s, are evolutionarily ancient and highly conserved families of proteins, found in all prokaryotes and eukaryotes, that are involved in protein folding, protection and transport. Their expression is increased when cells are exposed to elevated temperatures or other kinds of stress. HSPs can bind antigens and activate APCs. They can have a dual function, which means that they can be both primal (no need for the previous activation of APCs) and feedback (following the activation of APCs) molecules (Gallucci and Matzinger, 2001).

Immunological properties have been ascribed to several HSPs, including Hsp60, Hsp70, Hsp90, Gp96, and Calreticulin (Srivastava, 2002; Wallin et al., 2002; Osterloh and Breloer, 2008; De Maio, 2011). HSPs participate in the initiation of adaptive immune response by chaperoning peptide antigens for cross-presentation, they modulate PAMP-induced immune stimulation, and finally, in the absence of pathogen, they function as endogenous danger signals (Osterloh and Breloer, 2008).

An important concern is the possibility that the postulated pro-inflammatory function of HSPs might in fact be due to contamination with bacterial components, including LPS, even in allegedly “purified” settings (Wallin et al., 2002; Kono and Rock, 2008; Chen and Nuñez, 2010; Broere et al., 2011; van Eden et al., 2012). Even more importantly, several studies suggest that, depending on different contextual aspects, some HSPs can have a regulatory role, and not a proinflammatory one (van Eden et al., 2005; Quintana and Cohen, 2011). According to Shields et al., some HSPs (including HSP10 and HSP27) should be considered as “RAMPs” (resolution-associated molecular patterns), rather than “DAMPs” (damage-associated molecular patterns), as they seem to play a role in counterbalancing the consequences of a strong inflammatory response (Shields et al., 2011). The group of van Eden has recently offered a critique of the concept that HSPs should be seen as DAMPs; instead, according to these authors, they should be considered as “DAMPERs,” because they tend to have a regulatory effect on immune homeostasis (Broere et al., 2011; van Eden et al., 2012). Responding to Broere et al., Chen and Nuñez (2011) admit that “the evidence that HSPs act as DAMPs is weak at best.”

Finally, it is interesting to emphasize that, according to some studies on autoimmune diseases, a self–non-self distinction should be reintroduced to better explain how HSPs work. Indeed, based on investigations on autoimmune arthritis, it has been suggested that, during cellular stress, self-hsps are upregulated, generating a regulatory response that prevents or limits potential immunopathology caused by the stressors, whereas the foreign hsp65 generates an inflammatory pathogenic response after microbial challenge (Moudgil and Durai, 2008). Thus, even though HSPs have been considered as the paradigmatic example of DAMPs, no consensus has yet been reached on this claim.

### Necrotic cell death

Matzinger and colleagues oppose apoptotic (“normal,” “physiological”) cell death to necrotic (“abnormal,” “non-physiological”) cell death (Gallucci and Matzinger, 2001; Matzinger, 2002). Contrary to apoptotic cell death, necrotic cell death triggers “damage” signals. This idea rests on several observations. In mice it was shown that cells killed by necrotic death can activate resting DCs, in contrast with cells dying by apoptosis (Gallucci et al., 1999). In the case of humans, it was demonstrated that DCs are able to distinguish two types of tumor cell death, with necrosis providing a critical signal that will promote the initiation of immunity (Sauter et al., 2000). In addition, the demonstration that necrotic cells, but not apoptotic cells, can release HSPs, and that HSPs can activate DCs (Basu et al., 2000), strengthened this concept [though this idea is also criticized by van Eden et al. (2012)].

When cells die by necrosis, they lose the integrity of their plasma membrane and therefore they release their intracellular contents, including DAMPs that were up to that point hidden from immune receptors, in the extracellular matrix. Most of the time, this is not true for cells that die by apoptosis (though it can happen if they are not cleared rapidly). The release of DAMPs in the extracellular milieu functions as a sign of cell death to the innate immune system, which then triggers a pro-inflammatory response (Kono and Rock, 2008). This view, which elaborates on the danger theory, has been called the “hidden self model” (Kono and Rock, 2008).

In addition, analyses of immunogenicity associated with necrotic cell death include the case of eosinophils (Stenfeldt and Wennerås, 2004; Kobayashi et al., 2010): in human systems, eosinophilic granulocytes occur at interfaces with the external environment, e.g., the gastrointestinal, genitourinary, and respiratory tracts. Damaged epithelial cell lines derived from genital (HeLa cells), respiratory (HEp-2 cells) and intestinal tracts (HT29 cells) will cause eosinophilic migration, release of putative tissue-damaging factors, e.g., eosinophil peroxidase (EPO) and eosinophil cationic protein (ECP). Necrotic intestinal cells induced chemotaxis in human eosinophils.

### Recently described damage signals

Several “damage signals” or “DAMPs” have been described recently by various investigators (reviewed in Harris and Raucci, 2006; Bianchi, 2007; Kono and Rock, 2008). Although it is too soon to say whether they will be considered as genuine damage signals in the future, it is important to mention them here, if only to show the vitality of current discussions over the “danger theory” within the community of immunologists.

Before examining these signals, it must be emphasized that a significant progress in the field has been the recent attempt to offer criteria for establishing that a candidate molecule is a legitimate DAMP (Harris and Raucci, 2006; Bianchi, 2007; Kono and Rock, 2008; Matzinger, 2012). Kono and Rock suggest the four following criteria: (1) a DAMP should be active as a highly purified molecule; (2) the biological activity of a DAMP should not be due to contamination with microbial molecules (such as LPS, for instance); (3) a DAMP should be active at concentrations that are actually present in pathophysiological situations; (4) the selective elimination or inactivation of a DAMP should ideally inhibit the biological activity of dead cells *in vitro* and *in vivo* (Kono and Rock, 2008). As the authors admit, these criteria are rarely met by the presumptive DAMPs that have been described so far. Yet, they offer a guide for future research.

A first important example of recently identified damage signal is uric acid. Uric acid is involved in triggering immune responses, as it is released by injured cells, stimulates dendritic cell maturation and, when co-injected with antigen *in vivo*, significantly enhances the generation of responses from CD8^+^T cells (Shi et al., 2003). Shi et al. reveal that uric acid is an endogenous danger signal released by injured cells. More recently, the same group reported that elimination of uric acid reduced the generation of CTL to an Ag in transplanted syngeneic cells and the proliferation of autoreactive T cells in a transgenic diabetes model (Shi et al., 2006). From an evolutionary point of view, uric acid could represent a physiologic alarm signal gone awry in Western societies (Johnson et al., 2009). Together, these results support a molecular link between cell injury and immunity.

A second important example is High-mobility-group box 1 (HMGB1). HMGB1 has been described as a paradigmatic damage signal, because it is either secreted actively by inflammatory cells or released passively as a soluble molecule by necrotic (but not apoptotic) cells. It signals tissue injury and initiates the inflammatory response and/or repair (Harris and Raucci, 2006; Bianchi, 2007).

Third, the sensing of damage signals has been associated with the constitution of inflammasomes (Pedra et al., 2009; Chen and Nuñez, 2010). An inflammasome is a multiprotein complex that contains a pattern recognition receptor (PRR), typically a member of the Nucleotide-binding domain and Leucine-rich repeat containing Receptor (NLR) family. The inflammasome can activate Caspase 1, and consequently the production of IL-1β, playing an important role in the pro-inflammatory response (Chen and Nuñez, 2010). The NLRP3 (NOD-, LRR-, and pyrin-dolain-containing 3) inflammasome has been described as a “sensor” of immune danger signals (Cassel et al., 2009; Pedra et al., 2009). The NLRP3 inflammasome seems to be able to sense non-microbial molecules, or, in other words, it can be activated in the context of “sterile inflammation” (Chen and Nuñez, 2010), and it has been implicated in various sterile inflammatory diseases, including gout, asbestosis and silicosis. In a recent paper, Schroder et al. (2010) propose that interleukin-1 β (IL-1 β), reactive oxygen species (ROS), and thioredoxin-interacting protein (TXNIP) are all implicated in the pathogenesis of type 2 diabetes mellitus (T2DM). Because the NLRP3 inflammasome also drives IL-1 β maturation and secretion in gout, another disease of metabolic dysregulation, the authors propose that the NLRP3 inflammasome contributes to the pathogenesis of T2DM and gout by functioning as a sensor for metabolic stress. The role of NLRP3 inflammasome in a variety of metabolic diseases (including obesity, atherosclerosis and type 2 diabetes) is currently under investigation (De Nardo and Latz, 2011).

Other potentially significant examples of damage signals include IL-1 α; S100 proteins; hepatoma-derived growth factor (HDGF) [these three signals are reviewed in Bianchi (2007)]; high concentrations of adenosine 5′-triphosphatase (ATP) (Mariathasan et al., 2006; Riteau et al., 2010; Kouzaki et al., 2011) possibly in interaction with adenosine (Ado) (Bours et al., 2006); the release of different danger signals after the breakdown of extracellular matrix components, which has been involved in several diseases, such as pulmonary fibrosis (Riteau et al., 2010), and graft-versus-host disease (Zeiser et al., 2011); β-D-glucopyranosylceramide for the activation of invariant natural killer T cells (*i*NKT cells) (Brennan et al., 2011); IL-33, an alarmin released from necrotic cells and that acts directly on antiviral CD8^+^T cells (Bonilla et al., 2012); and F-Actin, an evolutionarily conserved DAMP recognized by DNGR-1, which is a receptor exposed by necrotic cells (Ahrens et al., 2012; Zhang et al., 2012). Recent research has also been done to gain new insights about the distinction between endogenous and bacterial DAMPs (Bianchi and Manfredi, 2009; Chen et al., 2009).

Finally, inflammation and immune responses may be triggered by modified danger signals. According to Lundberg and Yan, there are endogenous proatherogenic danger signals and corresponding molecular mechanisms of innate immune signaling in atherosclerosis (Lundberg and Yan, 2011). The identity and signaling mechanisms of LDL (low-density lipoprotein)-derived inflammatory components are central in understanding the pathogenic role of modified LDL in the etiology of atherosclerosis. LDL-derived phospholipids and cholesterol crystals act as endogenous danger signals that trigger Toll-like receptors and nucleotide binding oligomerization domain-like receptor inflammasome. Thus, they initiate inflammatory responses and promote disease progression. Clarity of the causal role of LDL in atherosclerosis offers a new approach to modified LDL-derived danger signals.

Turning now to a second, according to Sawamura et al. LOX-1 is a multiligand receptor associated with endothelial dysfunction and atherosclerosis; however it was first identified as an oxidized LDL receptor. LOX-1 is a unique molecule among those that sense danger signals. In addition to modified LDL and heat shock protein, LOX-1 scaffolds other sensors of danger including C-reactive protein and C1q. In this role LOX-1 is vital and commands responses to danger signals by acting as a cell adhesion molecule. By way of these functions, LOX-1 might function as a surveillance molecule important for the maintenance of vascular homeostasis (Sawamura et al., in press).

One of more unique presumptive modified danger signals may encompass views and usages of nanotechnology. According to Fadeel, nano associations have been long standing without recognizing their reasonably obvious role with respect to immune system triggering (Fadeel, 2012). After all, as he asserts, DNA is a nanoscale structure that contains the genetic code and the cell operates numerous nanoscale machines such as the proteasome (for degradation of proteins) and the ribosome (for protein synthesis). All these examples have led him to strongly advocate a closer look at modified nanoparticles and how they could interact with the immune system and whether they should be considered as danger signals capable of becoming NAMPS (nanoparticle-associated molecular patterns) that act as a newly identified “alarmin.”

## Immune responses without damages?

### The possibility of immune responses without damage

It is not clear whether damages *accompany* or *cause* immune responses. Some PAMPs can trigger an immune response with no accompanying damage (Vance, 2000). Because these PAMPs play an essential role in activation of innate immunity, they constitute an important objection to the danger theory, which states that every immune response is due to damages. Joffre et al. show that release of pro-inflammatory cytokines, is not sufficient to trigger a functional T-cell response, contradicting the danger theory (Joffre et al., 2009). Moreover, some grafts seem to trigger an immune response in the absence of danger (Bingaman et al., 2000). Finally, activation of regulatory T cells (or other regulatory mechanisms) is not supported by any satisfying explanation within the danger theory, as they do not seem to be triggered by inflammation or damages.

### In many cases damages are caused by the immune system itself

The immune system is often at the origin of tissue damages. If every immune response is caused by damage and every immune response causes damage, then the organism should enter into a vicious circle of immune activation, which is luckily not the case. In addition, the first pro-inflammatory signals are released by the “first line” of immune cells, due most often to macrophages. Thus, a reasonable question is: why have these cells been activated in the first place? They have not been activated by the damage itself (Vance, 2000). The danger theory seems here to confuse an effect of the immune response with its cause: in many cases, inflammation and tissue damage do indeed accompany an immune response, without provoking it.

## Danger and innate immunity: can the danger theory account for innate immunity?

Current immunologists tend to divide immunity between “innate” immunity and “adaptive” immunity, although the frontier between the two can be difficult to establish (Cooper et al., 2002; Cooper, 2008, 2010; Vivier et al., 2011). Innate immunity (in invertebrates and vertebrates) is considered to be *natural, non-specific, nonanticipatory*, and *non-clonal* but *germ-line* encoded, whereas *adaptive immunity* (in vertebrates) is defined as *induced*, *specific*, *anticipatory*, *clonal*, and *somatic*. There has been a growing interest in innate immunity in the last 20 years, provoking the question whether the danger theory has contributed to understanding innate immunity.

The initial danger theory was not intended to account for innate immunity. Significant is the fact that Matzinger herself was surprised to be asked by Charles Janeway what her theory could bring to the analysis of innate immune responses: “How would one compare the system of cells and molecules that make up the body's first line of defense against pathogens with a model that attempts to lay out the adaptive immune system's guidelines for immunity and tolerance?” (Matzinger, 1998: 399). The danger theory was first thought as an explanation for triggering lymphocytic immunity. Matzinger regularly refers to the “two signal” theory (Bretscher and Cohn, 1970; Lafferty and Cunningham, 1975). Though Janeway may be viewed as the true leader in this movement, Matzinger undoubtedly played a role in diffusing the idea that understanding activation of APCs was crucial (e.g., Gallucci et al., 1999). However, asking how lymphocytes are activated by APCs is, in fact, different from asking how innate immunity is triggered. Rather, it may simply be described as a renewed way to ask the question of what triggers an *adaptive* immune response.

Yet the danger theory may be able to explain innate immune responses. Long before immunologists opposed self–non-self and favoring danger, invertebrate immunologists invented or perhaps anticipated danger to help explain reactions to harmful agents. In 1988, Tina Trenczek first used the term “danger” as a component of injury and immunity in insects thus anticipating two mechanisms of initiating an invertebrate (and later vertebrate) immune response (Trenczek, 1988).

At least one example tends to support the danger theory in the domain of innate immunity in invertebrates. In the greater waxmoth *Galleria mellonella*, pathogens display both “microbe-associated molecular patterns” (MAMPs) and trigger danger signals, that stimulate a robust immune response, whose nature and strength may be determined by balancing danger and MAMP signals (Lazzaro and Rolff, 2011).

Thus, several observations suggest that innate immune mechanisms (in invertebrates but also in plants) may be triggered by damages done to the host (some of them have been described in section “From ‘danger’ to ‘damages’: The molecular identification of “damage signals” above). Seong and Matzinger suggested a damage-based framework that could unify innate and adaptive immune responses. According to this view, both PAMPs and endogenous danger signals belong to an evolutionarily conserved family of DAMPs that consist of exposed hydrophobic portions (“hyppos”) (Seong and Matzinger, 2004). Though this idea remains speculative (in particular because several PAMPs seem to trigger an immune response regardless of the emission of “damage” signals), it is stimulating to try to continue developing an integrative theoretical framework that would unify more clearly innate and adaptive immunity. Time will tell if the “damage” theory accomplishes this unification appropriately. For now, it is only possible to emphasize that it is a crucial challenge, one that still needs to be fulfilled.

An important concept for this challenge will be to take into account the organ-specificity of innate immune responses, that is, the fact that each organism may have its own way to regulate local innate immunity (Raz, 2007). More generally, context receives a growing attention among immunologists (Quintana and Cohen, 2011; Shields et al., 2011; see also Grossman and Paul, 2000; Zinkernagel, 2000; and Sumen et al., 2004, using intravital microscopy, or IVM). This idea of context-dependent immunity is consistent with the emphasis of danger theory proponents on the crucial role of tissues in immunity (Matzinger, 2007, 2012; Matzinger and Kamala, 2011), but integrating this idea fully will demand further experimental work.

## Danger and cancers

Initially, Matzinger and colleagues proposed that there was no immune response to tumors, and that it was well explained by the danger theory (Matzinger, 1998, 2002). They claimed that the danger theory proved its superiority to other theories in that only the danger theory could explain why there is no immune response to tumors. According to Matzinger (1998), “The Danger model suggests that no immune response occurs because tumors are healthy, growing cells that do not normally die necrotically or send out alarm signals.” Numerous recent data show that this view is inadequate (e.g., Dunn et al., 2006): the immune system does respond to tumors and eliminates many.

Can the danger theory be “saved” with regard to immunity to tumors by suggesting that, after all, tumors are dangerous and may well send “alarm signals?” The immune surveillance hypothesis can be reframed to accommodate the idea that tumors are immunogenic because they send alarm signals (though this idea is in complete contradiction with Matzinger's own view) (Dunn et al., 2006). Pastor-Pareja et al. (2008) present an example of a possibly damage-based innate immune response to tumors in the fruit fly *Drosophila melanogaster*. They show that tissue damage activates JNK signaling in both tumors and aseptic wounds, and this causes expression of JAK/STAT-activating cytokines. Later, the response cascades so that cytokine secretion occurs later from injured tissues that are then amplified into a systemic response by the induction of additional cytokine expression in hemocytes and in the fat body. Then, finally, there is proliferation of hemocytes. These results reflect important implications since they suggest common mechanisms in response to tumors and wounds in flies.

Therefore, it cannot be excluded that immune response to tumors are due to the emission of damage signals. Yet, tumor immunogenicity seems to be due to molecular modifications rather than damage *per se* (Pradeu and Carosella, 2006). Reframing the danger theory may then contradict explicitly the convictions of its proponents (Matzinger, 1998), thereby exposing all too clearly the excessive plasticity of the concept of “damage.”

## Danger and grafts

The explanation given by the danger theory concerning immune responses to grafts is not convincing. Matzinger (1994, 2002) asserts that immune responses against transplants are due to surgical damages. If it were true, how would it be that a surgical autograft is not followed by an immune rejection? Furthermore, the immune system seems to be able to respond to grafts in the absence of danger (Bingaman et al., 2000). Finally, how is it that transplant rejection occurs in nature (without surgical intervention), as in the case of rejection reactions between two protochordate colonies of *Botryllus schlosseri* for example (De Tomaso et al., 2005)?

## Danger and the tolerance of symbiotic exogenous entities

The danger theory claims that symbiotic exogenous entities, for instance symbiotic gut bacteria, are tolerated because they do not provoke damages. But this statement oversimplifies what an “immune response” is. Gut bacteria are not simply “tolerated” in the sense that they would not trigger any immune response; rather, the host and these bacteria continuously establish an equilibrium, in which the gut immune system does in fact respond to bacteria, but in a highly controlled way (Eberl, 2010). In addition, some bacteria “under control” can trigger strong immune responses in some circumstances, for example when they change their location in the intestine, even in the absence of damage.

## Conclusion

Thus, supporting data are two-pronged, some support immune responses to endogenous signals and even sometimes to damage signals, whereas other tend to show that damages are not always the *cause* of an immune response, casting doubts on the validity of the danger theory as a general, unified framework for immunity (Pradeu, 2012). Table 3 sums up the advantages and drawbacks of the danger theory that we presented.

**Table 3:** **Advantages and drawbacks of the danger (or “damage”) theory**.

**Immunological question**	**Assessment**
Criterion of immunogenicity	Satisfying
Importance of APCs in adaptive immune responses	
Immune responses to endogenous constituents	
Innate immunity	Not satisfying
Immune responses to tumors	
Immune responses to grafts	
Immune responses to symbiotic bacteria	

A key question is to determine whether the danger theory offers an adequate *explanation* for triggering immune responses. In several cases it is rather an *a posteriori* description, exposing the whole framework to a risk of tautology. The idea in this case is close to this: if an immune response did occur, then it is most likely due to the perception of “danger” somewhere. It should also be emphasized that proponents of the danger theory offered several important experimental mistakes, e.g., (1) no immune response to cancers, (2) immune responses to transplantation were caused by the surgeon's hand. These errors do not invalidate the general principle of the theory, but they may raise some suspicion. Moreover, when the theory is modified to account for new data or updated views (as in the case of immunosurveillance, for instance), flexibility of the language of “danger” seems problematic. Perhaps the danger theory can constitute a unified theory of immunity only at the cost of *ad hoc* hypotheses—something that its proponents wanted explicitly to avoid (Matzinger, 1998).

### Conflict of interest statement

The authors declare that the research was conducted in the absence of any commercial or financial relationships that could be construed as a potential conflict of interest.
